# Influence of surface-modified maghemite nanoparticles on in vitro survival of human stem cells

**DOI:** 10.3762/bjnano.5.183

**Published:** 2014-10-08

**Authors:** Michal Babič, Daniel Horák, Lyubov L Lukash, Tetiana A Ruban, Yurii N Kolomiets, Svitlana P Shpylova, Oksana A Grypych

**Affiliations:** 1 Institute of Macromolecular Chemistry, Academy of Sciences of the Czech Republic, Heyrovského nám. 2, 162 06 Prague 6, Czech Republic; 2 Department of Human Genetics of Institute of Molecular Biology and Genetics, NAS of Ukraine, Zabolotnogo 150, 03143 Kiev, Ukraine

**Keywords:** maghemite, magnetic, MTT assay, nanoparticles, stem cells

## Abstract

Surface-modified maghemite (γ-Fe_2_O_3_) nanoparticles were obtained by using a conventional precipitation method and coated with D-mannose and poly(*N*,*N*-dimethylacrylamide). Both the initial and the modified particles were characterized by transmission electron microscopy and dynamic light scattering with regard to morphology, particle size and polydispersity. In vitro survival of human stem cells was then investigated by using the methyl thiazolyl tetrazolium (MTT) assay, which showed that D-mannose- and poly(*N*,*N*-dimethylacrylamide)-coated γ-Fe_2_O_3_ particles exhibit much lower level of cytotoxicity than the non-coated γ-Fe_2_O_3_.

## Introduction

One of the most important applications of nanoparticles in biomedicine is the direct labeling of cells in order to track them both in diagnostics and therapeutics [1–2]. For example, mesenchymal [3], neural [4], and bone marrow [5] stem cells, as well as other cells are widely labeled by surface-coated iron oxide nanoparticles. Other applications of nanoparticles involve the delivery of drugs to specific types of cells in a body, in order to greatly reduce both the needed dosage and the side effects of the drug [6–8]. At the same time, the long-term fate of the particles and their possible cytotoxic effects on cells of the human body have to be taken into account to evaluate potential risks and side effects associated with use of such materials. In diagnostic and/or therapeutic applications, the presence of nanoparticles within the cells might not necessarily negatively influence their viability.

A variety of particles with sizes ranging from ten to hundred nanometers are used for the above mentioned purposes [9]. Monosized iron oxide nanoparticles, sometimes called ultra-small superparamagnetic iron oxide nanoparticles, play the dominant role. Quantum dots, gold and, recently, also upconversion nanoparticles are used less frequently. The main advantages of iron oxides (magnetite Fe_3_O_4_ or maghemite γ-Fe_2_O_3_) are their simple preparation and their magnetic properties, which are necessary for detection. Moreover, it is convenient that iron oxides are readily metabolized in the body. From this point of view, quantum dots are disqualified due to their toxicity.

Like in every biological application of foreign objects, the surface of the nanoparticles has to be coated by a biocompatible shell to prevent undesirable interactions of particles with the environment and to enable their internalization by the cells. At the same time coating avoids particle aggregation. Last but not least, the surface shell of the magnetic cores has to participate actively in the uptake of the conjugates, proteins and/or antibodies. Internalization (transfection) agents [10] or specific targeting groups [11–12] are therefore often bound to the particles in order to support their uptake by the cells. Surface modifications are already well described as the particles are used in many applications, such as magnetic contrast agents, separations, diagnostics, drug delivery, and hyperthermia [13–17]. In terms of coating, many low- and high-molecular-weight compounds were proposed, e.g., dextran [18–19] (in Feridex^®^ and Endorem^®^ developed as contrast agents for magnetic resonance imaging, MRI), poly(ethylene glycol) (PEG) [1], poly(*N*,*N*-dimethylacrylamide) (PDMAAm) [20], poly(L-lysine) [21–22], protamine sulfate [23], or layer-by-layer polyelectrolyte complexes [24].

The aim of this report is to describe the labeling of human fibroblast-like cells with new surface-modified superparamagnetic maghemite nanoparticles both before and after their surface coating with D-mannose or poly(*N*,*N*-dimethylacrylamide) and to determine the survival of the cells. Possible cytotoxic effects of the cells in contact with the nanoparticles are also discussed.

## Experimental

### Materials

Dimethyl sulfoxide (DMSO), 3-(4,5-dimethylthiazol-2-yl)-2,5 diphenyltetrazolium bromide (MTT), Thiazine Red (ThR) and 4',6-diamidino-2-phenylindole (DAPI) were purchased from Sigma-Aldrich (St. Louis, MO, USA); Dulbecco’s modified Eagle’s medium (DMEM) was from PAA Laboratories (Pasching, Austria). Non-coated, D-mannose- and poly(*N*,*N*-dimethylacrylamide)-coated γ-Fe_2_O_3_ nanoparticles (4.4 mg/mL) were prepared through coprecipitation of FeCl_2_ and FeCl_3_ solutions with ammonia, the subsequent oxidation of the resulting product by sodium hypochlorite and the coating with D-mannose and poly(*N*,*N*-dimethylacrylamide) according to earlier reports [20,25]. While coating with D-mannose was performed by the slow addition of D-mannose solution to the γ-Fe_2_O_3_ colloid, coating with PDMAAm included solution radical polymerization of *N*,*N*-dimethylacrylamide (DMAAm) in the presence of maghemite nanoparticles using 4,4'-azobis(4-cyanovaleric acid) initiator. The particles were examined with a JEOL JEM 200 CX transmission electron microscope (TEM; Tokyo, Japan) to determine the particle size and polydispersity; at least 500 particles were measured by using the Atlas software (Tescan Digital Microscopy Imaging, Brno, Czech Republic).

#### Cell culture and MTT test

The human established stem cell line 4BL originated from peripheral blood of a healthy donor was obtained from the Department of Human Genetics of the Institute of Molecular Biology and Genetics (NAS of Ukraine, Kiev). The cells were cultivated in standard DMEM medium with the addition of 100 U/mL penicillin, 100 µg streptomycin/mL and 10% fetal calf serum and reseeded to subconfluent state. The cells were used after long-time cultivation in vitro (more than one hundred passages). A microcultural MTT test was used for the estimation of the number of metabolizing cells both in control and in culture containing non-coated and surface-modified γ-Fe_2_O_3_ particles.

In a 96-well plate without surface treatment, 4BL cells were cultivated in standard DMEM medium with 10% fetal serum for 24 h and a series of aqueous γ-Fe_2_O_3_ colloids were added to reach concentrations of 100, 50, 25, 12.5 and 6 µL colloid per mL of fresh cultural medium. The cells were grown with the nanoparticles for 72 h until a confluent monolayer of the cells was obtained. Consequently, 15 μL of MTT dye (5 mg/mL) was added per well. Crystals formed inside the cells were dissolved by DMSO and the solution was introduced to the culture medium. The optical density of the culture medium in the wells was measured by using a MR 700 Microplate Reader (Dynatech; Sussex, UK) at 570 nm. The number of living cells (in terms of optical density) in cultures in presence and absence of the particles (control) were compared.

#### Fluorescence microscopy

Polypeptides of cell cytoplasm were stained with 0.001% ThR aqueous solution for 2–5 min and observed by fluorescence microscopy under excitation at 510 nm and emission at 580 nm [26]. For the visualization of nuclei in the cells, DNA-specific fluorescent DAPI dye was used under excitation at 350 nm and emission at 470 nm [27]. Anti-bleach reagent was used according to Johnson et al. [28]. The cells were investigated by using a CarlZeiss LSM 510 confocal laser scanning microscope (Carl Zeiss Oberkochen, Germany).

## Results and Discussion

### Surface-modified γ-Fe_2_O_3_ particles

**Figure 1:**
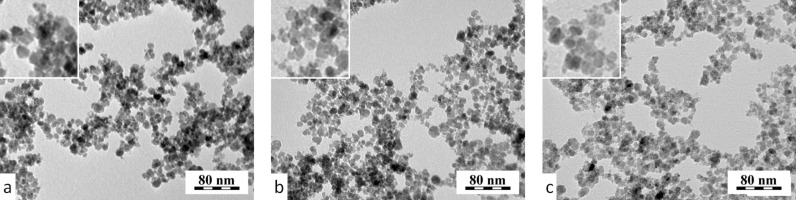
Transmission electron micrographs of (a) non-coated, (b) D-mannose- and (c) PDMAAm-coated γ-Fe_2_O_3_ nanoparticles.

In this report, non-coated γ-Fe_2_O_3_ nanoparticles served both as a control and as a core for post-synthesis coating with D-mannose and PDMAAm. TEM images of the synthesized magnetic particles did not substantially differ showing a relatively high uniformity in terms of size and spherical shape (Figure 1).

The average diameter of the particles was 6–7 nm and their polydispersity (weight- to number-average particle diameter) was 1.3–1.5 indicating a moderately broad particle size distribution. It should be pointed out that the particle size determined by TEM was smaller compared with the size measured by dynamic light scattering. The hydrodynamic size in water was in the range of 50–170 nm. The presence of the coating on the surface of the particles was confirmed by FT-IR spectroscopy [20,25]. In particular, PDMAAm-coated γ-Fe_2_O_3_ nanoparticles exhibited a long-term colloidal stability even after more than six months of storage. All types of γ-Fe_2_O_3_ nanoparticles displayed superparamagnetic behavior [29], which is characterized by a strong response to a magnetic field and zero remanent magnetization. This is proven by the quick separation of such particles in a magnetic field and the easy redispersion by Brownian motion into a liquid medium after removing the magnet. It is obvious that the colloidal stability of the particles is a prerequisite to their biomedical applications. Thanks to the zero remanent magnetization of the particles, the risk of formation of aggregates in physiological liquids is reduced.

### MTT assay

In order to achieve an efficient cell labeling, the response of intracellular γ-Fe_2_O_3_ content on the metabolism of the cells should be taken into account because the intracellular overload may cause cytotoxicity due to formation of free radicals. The cytotoxicity of non-coated, D-mannose- and PDMAAm-coated γ-Fe_2_O_3_ nanoparticles was evaluated by using a MTT assay with 4BL human cells. The assay is dependent on the ability of viable cells to metabolise a water-soluble tetrazolium salt into a water-insoluble formazan. DMSO is the best solvent for dissolving the formazan, especially if a significant amount of residual medium is left in the wells of the microtiter tray used for the assay. A reaction occurs between medium and formazan, which is accompanied with a change of the shape of the absorbance spectrum of the solution. When cells are incubated with MTT, the resulting optical density of the formazan product is dependent upon both the concentration of MTT and the incubation time. The optical density is stable for several hours after dissolution of the formazan in DMSO [30].

**Figure 2:**
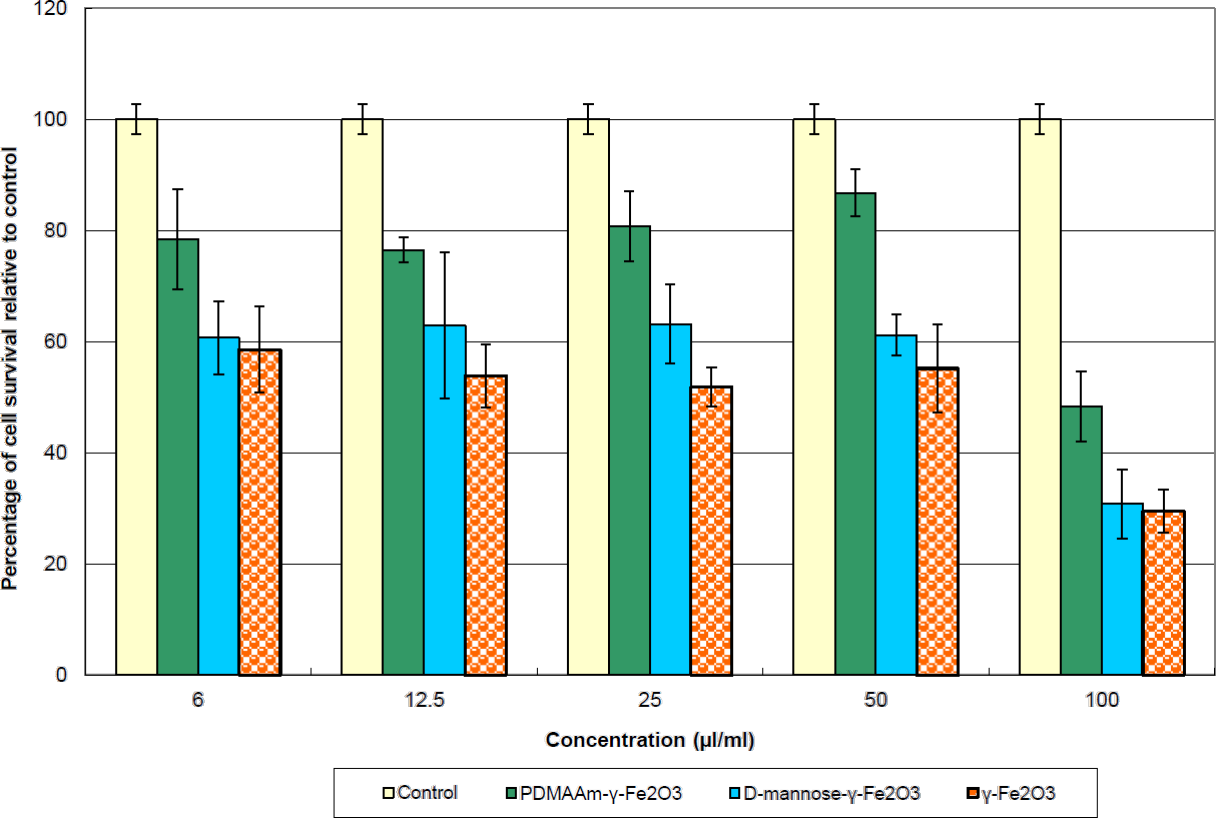
Effect of non-coated, D-mannose- and PDMAAm-coated γ-Fe_2_O_3_ on the in vitro survival of 4BL human cells at different concentrations of particles in the medium.

The concentration-dependent effect of D-mannose- and PDMAAm-coated γ-Fe_2_O_3_ nanoparticles on the cell viability was determined after incubation for 72 h and compared with the cells in the absence of nanoparticles (control experiment). All investigated γ-Fe_2_O_3_ nanoparticles exhibited a cytotoxic influence on human cells in vitro (Figure 2). The optical density of all cell cultures treated with the nanoparticles differed from the control with *p* < 0.05 calculated by a two-sample t-test. Statistically significant differences were obtained for all investigated types of the particles compared with the control and all of them revealed cytotoxic activity. At the same time, the data confirmed the tendency of decreasing particle cytotoxicity if coated with PDMAAm or D-mannose.

**Figure 3:**
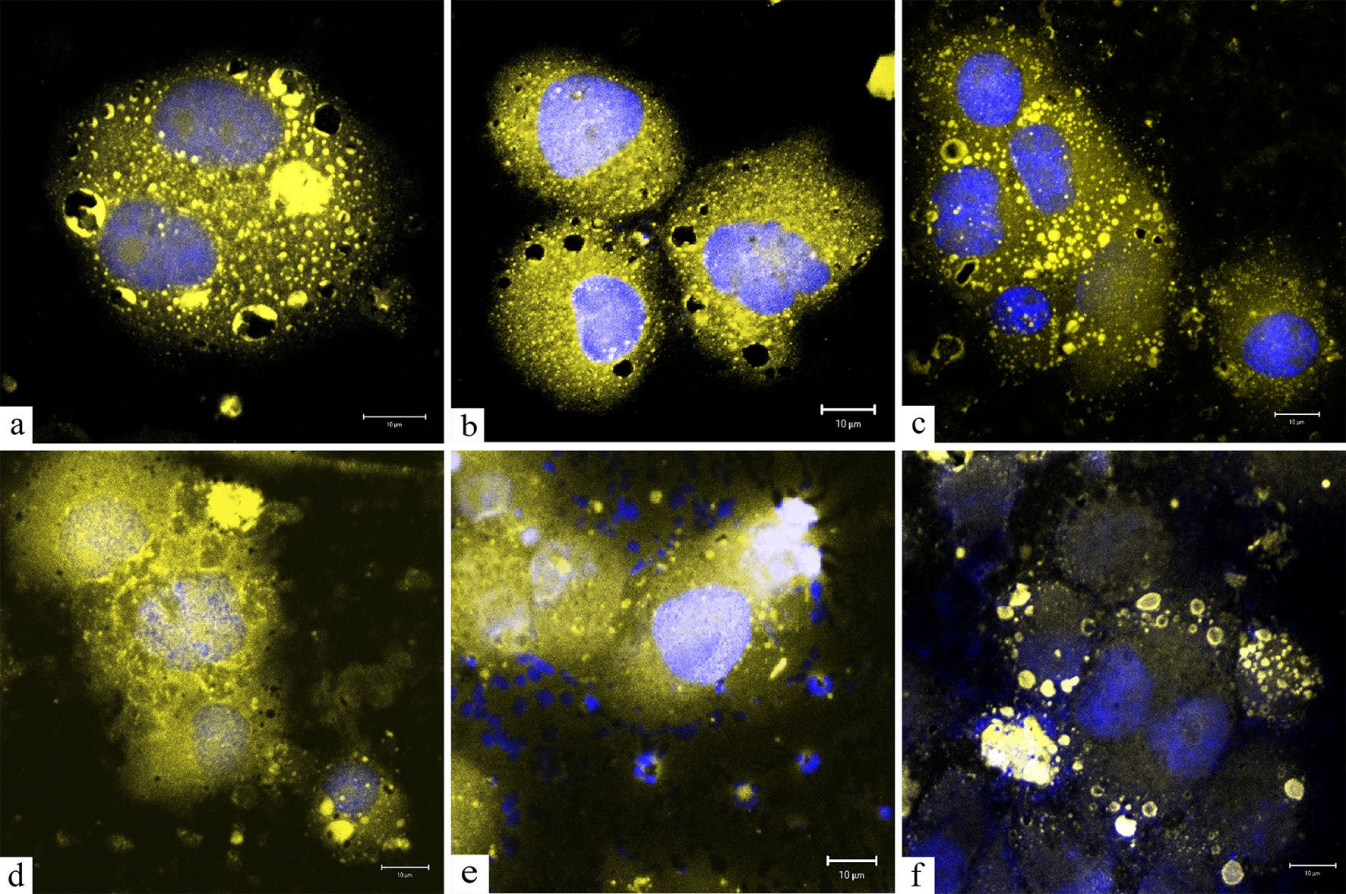
Confocal micrographs of 4BL human stem cells treated with (a, b) D-mannose-coated γ-Fe_2_O_3_, (c, d) PDMAAm-coated γ-Fe_2_O_3_ and (e, f) non-coated γ-Fe_2_O_3_ nanoparticles. Staining with DAPI and ThR. Scale bars: 10 μm.

The strongest cytotoxic effect was observed at the highest concentration of the colloid (100 µL/mL of medium). The PDMAAm-coated γ-Fe_2_O_3_ nanoparticles showed a lower cytotoxicity at all concentrations (statistically significant results) compared with D-mannose-coated and non-coated γ-Fe_2_O_3_ nanoparticles. D-mannose-coated nanoparticles were less cytotoxic at concentrations of 50, 25 and 12.5 μL γ-Fe_2_O_3_ colloid/mL than non-coated γ-Fe_2_O_3_. The beneficial effect of coating (diminishing particle cytotoxic activity for the cells) was thus demonstrated. By using confocal microscopy, D-mannose- and PDMAAm-coated γ-Fe_2_O_3_ nanoparticles were detected in vacuoles inside the cytoplasm of the cells (Figure 3a,c). PDMAAm-coated particles were occasionally observed outside the surface of the cellular membrane (Figure 3d). The structures appearing as dark holes in the cytoplasm are believed to be vacuoles completely filled with D-mannose-coated γ-Fe_2_O_3_ (Figure 3b). However, non-coated γ-Fe_2_O_3_ particles inside the cells were not observed (Figure 3e), although many cells were destroyed after treatment with the nanoparticles (Figure 3f). Obviously, unmodified γ-Fe_2_O_3_ particles were not internalized by the cells and could be responsible for cell death. The influence of iron oxide nanoparticles on the morphology of the vital organs of mice after unitary intravenous introduction of a nanoparticle colloid was described earlier [31–33]. This confirmed adaptive reactions of the mouse organism. Morphological changes of the organ cells may result from the direct action of nanoparticles on the cells, or may also indirectly result from impaired microcirculation, the activation of plasma protein systems and the release of cellular mediators that cause ischemic, toxic or receptor-mediated cell damages. In this regard, future attention should be focused on remote consequences of intravenous introduction of the nanoparticles.

## Conclusion

In order to increase the cellular uptake of the magnetic nanoparticles and enhance their specific targeting effect, surface functionalization has to be employed to coat the nanoparticle surface with ligands that could specifically interact with the receptors overexpressed in the cell membrane. While the size of the dry nanoparticles was 6–7 nm according to TEM, the hydrodynamic diameter in water was more an order of magnitude larger due to partial agglomeration and the different nature of the measurements. Nevertheless, the particles formed stable colloidal solutions. The developed D-mannose- and PDMAAm-coated γ-Fe_2_O_3_ particles were found to have a reduced cytotoxic activity compared to non-coated nanoparticles, which was demonstrated by the methyl thiazolyl tetrazolium (MTT) assay. However, all γ-Fe_2_O_3_ particles tested at different concentrations reduced the viability of human cells in vitro. It should be noted that only D-mannose- and PDMAAm-coated γ-Fe_2_O_3_ particles were internalized by the cells and subsequently found then in the cytoplasm. These nanoparticles can thus serve as potential probes for cell imaging. In particular, PDMAAm proved to be a highly efficient coating providing several attractive properties. These include high hydrophilicity, easy introduction of functional comonomers by copolymerization and the possibility to control both the molecular weight and the thickness of the shell. PDMAAm-coated γ-Fe_2_O_3_ particles seem to be thus a perspective basis of advanced core–shell architectures, e.g., for stealth particles with reduced opsonization in biological fluids. These properties can be exploited in magnetic resonance imaging and tracking of iron oxide-labeled cells, for the magnetic separation of cells, nucleic acids and proteins and in medicine for treatments by using targeted drug delivery, magnetic hyperthermia or magnetofection.
